# Strong Coupling of Organic Molecules 2023 (SCOM23)

**DOI:** 10.1515/nanoph-2024-0260

**Published:** 2024-05-27

**Authors:** Joel Yuen-Zhou, Wei Xiong

**Affiliations:** Department of Chemistry and Biochemistry, 8784 University of California San Diego, La Jolla, CA, 92093, USA

On June 20–22 2023, the international conference Strong Coupling of Organic Molecules (SCOM23) was held in La Jolla, California, in the campus of the University of California San Diego. This 4th edition of SCOM is a follow-up of earlier successful editions in San Sebastián (2016), Eindhoven (2018), and Gothenburg (2021, virtual). SCOM serves as a vibrant and interdisciplinary meeting ground for physicists, chemists, material scientists, and engineers, with the common goal of identifying and elucidating novel phenomena involving molecular polaritons, hybrid light–matter modes resulting from the strong coupling of micro- and nano-confined photon modes with material excitations in molecular ensembles or materials. This special issue of *Nanophotonics* collects contributions from many participants of SCOM23 and beyond, and serves as a testament of the timeliness of the field as well as its breadth of scope and opportunities.

From a historical perspective, the discovery of the hybridization of light and material excitations in crystalline solids outside of cavities can be traced back to the seminal works of Tolpygo [1] and Huang [2] on phonon-polaritons and of Agranovich [3] and Hopfield [4] on exciton-polaritons in the 1950s; as far as we are aware, the latter paper is the responsible for coining the term “polariton.” It was however, until the 1992, when Weisbuch [5] demonstrated that these strong coupling phenomena in inorganic semiconductors could be significantly enhanced with optical microcavities. A few years later, Lidzey [6] reported the same feat with excitons in disordered organic films. Much attention in the polaritonics community initially focused on many-body physics phenomena such as polariton lasing [7], [8] and condensation [9], [10]; however, approximately within the last decade, an effort primarily spearheaded by the Ebbesen group in Strasbourg has demonstrated that polariton formation may lead to a richer class of phenomena, including modified electronic ground and excited state chemical kinetics and dynamics [11], creating much excitement through an emergent new field of research that has been broadly known as “polariton chemistry.” [12] The possibility that material degrees of freedom could be affected in unique ways by strong light–matter coupling (even in vacuum, in the absence of photons [13]) is of course tantalizing and has catalyzed much activity in the field, although many puzzles still remain about the mechanisms whereby these modifications occur. These explorations have also energized efforts to understand the dynamics of molecular polaritons via optical spectroscopy, and more broadly, to harness molecular polaritons for photonics, optoelectronic applications, and quantum simulation and sensing [14], [15], [16].

While the main emphasis in the SCOM community is on strong coupling of photons with molecular materials in the optical and infrared frequency regime, much can be learned from analogous studies in solid-state materials or even in other setups supporting AC dipoles through long wavelength excitations. For instance, in this issue, Meng et al. [17] provide a concise overview of current architectures that showcase strong coupling between Fabry–Perot cavities and THz resonances realized with artificial atoms based on plasmonic metamaterials. Given that the natural timescales and lengthscales of these systems are a few orders of magnitude longer than the typical studies involving vibrational and electronic excitations in molecules, THz polaritonics provides a convenient playground to explore novel strong coupling phenomena in conceptually simpler setups, while simultaneously shedding light on similar possibilities for future experiments with molecular degrees of freedom.

Several contributions in this issue correspond to studies in the linear optics of molecular polaritons. A unifying theme in some of these studies is the fact that the bulk linear optical properties of a molecular ensemble alone outside of a cavity are enough to determine the linear optical properties of the resulting polaritons in the collective strong coupling [18], [19]. In particular, Rider et al. [20] show us how using molecular absorption properties one can screen for materials that are guaranteed to showcase large Rabi splittings. Schwennicke and Yuen-Zhou [21] use it to provide a prescription to extract collective coupling intensities from disordered ensembles (a ubiquitous scenario in molecular materials) given information from linear absorption, reflection, and transmission spectra. These classical optics treatments are consistent with quantum formalisms involving large dipole ensembles, such as in the non-Markovian quantum state diffusion method described by Leppälä et al. [22], which successfully treats complex molecular lineshapes.

Other articles in this issue report novel linear optical effects involving polaritons. For instance, Kędziora et al. [23] report an unconventional Fabry–Perot cavity containing a thin perovskite film but also an air gap; this setup serves as a playground for non-Hermitian physics and endows the linear optical properties of the system with non-Hermiticities that translate into asymmetric linear optical responses depending on whether the cavity is probed from one mirror or from the other. García et al. [24] demonstrate the onset of the ultrastrong coupling regime for an architecture consisting of the very bright collective dipole ensemble of self-assembled Au nanoparticles interacting with a p-NiO/Au film which supports a Fabry–Perot mode. Furthermore, Tserkezis et al. [25] demonstrate the possibility of cavity-free hybridization between interband transitions and Mie resonances by exploiting the high-index contrast between inorganic semiconductor nanocylinders and their surroundings.

While the examples above demonstrate a reasonably good agreement theory and experiment when dealing with linear optical properties of molecular polaritons, nonlinear optical counterparts seem to be a frontier of research that is not fully solved yet. Sufrin et al. [26] report the first experimental double-quantum coherence spectroscopy study of vibrational polaritons of CO stretches of PMMA coupled to infrared surface lattice resonances. Their study is a sensitive probe for molecular anharmonicities, and reveals that vibrational polaritons feature a much smaller anharmonic shift compared to their bare molecular counterparts, in consistency with previous theoretical work [27] and experimental works of inorganic semiconductor quantum-well exciton polaritons [28]; however, work must be carried out to accurately predict the measured transition intensities, which do not agree with previous theory.

Another important frontier in molecular polaritonics is the exploration of whether photophysical processes in organic materials can be modified under strong coupling. Promising results are reported in a number of contributions concerning energy transfer and transport. For instance, de Jong et al. [29] who report enhanced internal quantum efficiencies in an organic photovoltaic setup featuring polariton formation involving excitons in the donor-acceptor heterojunction blend, along with a reduction of the Urbach energy. Cargioli et al. [30] show active control of polariton-assisted remote energy transfer using molecular photoswitches that can change their absorption spectrum with an optical pulse [31], [32] and hence, turn this transfer on and off in an ultrafast timescale. In a similar vein, Sokolovskii and Groenhof [33] theoretically and computationally demonstrate that polariton transport of energy can be switched on with a pulse triggering a photochemical reaction (photoacid dissociation). Aroeira et al. [34] provide an analytical theory to understand the role of exciton transport assisted by polaritons in multimode cavities; they find that for small amounts of disorder, this transport is strongly dependent on the initial excitonic composition as well as on the momentum of the wavepacket prepared by the laser, while for strong amounts of disorder this initial state is almost inconsequential.

The photophysical phenomena mentioned in the previous paragraph involves intermolecular energy transfer processes where much evidence points towards polaritons being useful for their control [35], [36]. A perhaps more challenging goal is the control of intramolecular photophysical processes such as photoluminescence kinetics. In one study, when coupling to a multimode cavity, Odewale et al. [37] demonstrate an intriguing photoluminescence enhancement (beyond the typical Purcell effect) of copper(II) tetraphenyl porphyrin molecules under strong coupling due to a putative concomitant enhancement of non-Condon coupling between different electronic states. In another study, however, Abdelmajid et al. [38] subject an OLED film to strong coupling with the excitonic transitions of a material that undergoes thermally-assisted delayed fluorescence due to the recombination of trapped charges, and conclusively demonstrate that no changes in the photoluminescence kinetics are observed when compared to the bare film. The discrepancies in these two studies highlight the subtlety of polaritonic effects, which merit further exploration. New techniques for model building might be important to make progress in this topic, prompting Wu et al. [39] to develop an interesting transfer tensor method to collect short-time information from a polaritonic experiment or simulation, and from there, systematically infer long-time dynamical information.

Another active topic in the SCOM community is vibropolaritonic chemistry [13], the effort whereby strong coupling between infrared microcavity modes and ensembles of high-frequency molecular vibrations gives rise to changes in electronic ground-state reaction kinetics, even in the absence of external optical pumping. Many experimental and theoretical efforts trying to understand these effects have faced challenges, and there is no consensus as to their mechanisms [40], [41]. In fact, Chen et al. [42] report the experimental study of hydrogen abstraction reactions by CN radicals in cyclohexane/chloroform solution; subjecting the CH stretching mode of cyclohexane to vibrational strong coupling over a wide range of Rabi splittings, they do not identify any appreciable effects of the strong coupling in the observed chemical kinetics when compared to out-of-cavity benchmarks, suggesting not all reactions can be modified by polaritons [43], [44]. Ying et al. [45] provide a theory that shows that strong coupling of the *k* = 0 mode of a Fabry–Perot cavity to the vibration of a molecule can induce enhancement of a symmetric double-well tunneling reaction; however, this effect washes out in the collective strong coupling regime, which is the regime at which reactions operate. Finally, Lindloy et al. [46] carry out rigorous computational studies for two model reactions (a symmetric double-well barrier crossing and a proton transfer) under vibrational strong coupling with a single-mode cavity using new tools based on the hierarchical equations of motion (HEOM); they run finite-temperature simulations with a few molecules in a cavity and use a mean-field approach to reach the thermodynamic limit of collective strong coupling. For the few molecule studies, they observe that cavity-induced dissipation can enhance reactivity in the Kramers underdamped regime, but that this effect washes out in the collective regime, unless nonequilibrium initial conditions are considered, agreeing with recent experimental observations [47]. A few intriguing suggestions are proposed as possible avenues to try for solving the vibropolaritonic chemistry puzzle hereafter: the inclusion of multimode effects (as in Ying’s study), of the spatial profile of the electric field of the cavity modes, and of intermolecular interactions.

One of the most interesting features of molecular polaritons is that, unlike their inorganic semiconductor counterparts, they can achieve condensation at room temperature. However, the processes that dominate the thermalization of these molecular polariton condensates are not fully understood, and are dominated by vibronic couplings that are much stronger than in molecules than in the solid state. Tereshchenkov et al. [48] develop a theoretical model to understand the molecular parameters that control thermalization and thus efficient condensation of molecular polaritons. An application of these condensates is the room-temperature simulation of many-body physics; for instance, Peng et al. [49] experimentally demonstrate that the repulsive interactions between the condensates in one- and two-dimensional arrays of organic-inorganic perovskite microcavities can simulate XY spin Hamiltonians. Another application is provided by Lempicka et al. [50], who show that perovskite condensates in cavities supplemented with a birefringent nematic liquid crystal can give rise to coherent light emission whose frequency and polarization (linear, circular) can be electrically tuned on-demand.

Finally, the onset of strong coupling with few molecules (instead of the standard collective regime in microcavities) is poised to enhance polariton chemistry effects. This comes at the expense of reduced small volumes that are found in complex nanophotonic environments and therefore require the development of new theoretical methodologies. Along these lines, Sánchez-Martínez et al. [51] provide a simple and accurate way to derive quantum master equations for emitters coupled to arbitrary photonic environments; the strategy is to divide the spectral density of the later into resonant modes that couple strongly with the emitter and the rest, which can be treated as a Markovian bath. In another development, Ojambati [52] experimentally demonstrate and theoretically interpret a version of nanolasing with 2 to 50 methylene-blue emitters embedded in nanocavities (nanoparticle on a mirror architectures) in the weak coupling regime; while spectral narrowing is not observed, enhanced coherence and directionality of emission is measured.

This collection of articles provides a good overview of the status of the field and we hope it serves as a guide and inspiration for incoming researchers. Newcomers to the field will recognize that the field provides much opportunity to ask novel questions in photonics but with a twist that involves accounting for nonequilibrium processes of molecular matter, and which therefore necessitates theoretical and experimental techniques that interpolate between molecular and photonic length/time scales. Because of this, SCOM is a burgeoning field that is poised to grow in upcoming years. We thank the Nanophotonics Publishing editor Dennis Couwenberg and publishing assistant Tara Dorrian for support in the preparation of this collection.
